# The development of a guideline implementability tool (GUIDE-IT): a qualitative study of family physician perspectives

**DOI:** 10.1186/1471-2296-15-19

**Published:** 2014-01-29

**Authors:** Monika Kastner, Elizabeth Estey, Leigh Hayden, Ananda Chatterjee, Agnes Grudniewicz, Ian D Graham, Onil Bhattacharyya

**Affiliations:** 1 Li Ka Shing Knowledge Institute of St. Michael’s Hospital, 209 Victoria Street, Toronto, Ontario, Canada; 2 Strategic Policy, Planning & Initiatives, Health Services, Region of Peel, Toronto, Ontario, Canada; 3 University of Ottawa, Ottawa Hospital Research Institute, Ottawa, Ontario, Canada; 4 Department of Medicine, Faculty of Medicine, University of Toronto, Toronto, Ontario, Canada

**Keywords:** Guideline implementability, Family practice, Knowledge translation, Qualitative

## Abstract

**Background:**

The potential of clinical practice guidelines has not been realized due to inconsistent adoption in clinical practice. Optimising intrinsic characteristics of guidelines (e.g., its wording and format) that are associated with uptake (as perceived by their end users) may have potential. Using findings from a realist review on guideline uptake and consultation with experts in guideline development, we designed a *conceptual* version of a future tool called Guideline Implementability Tool (GUIDE-IT). The tool will aim to involve family physicians in the guideline development process by providing a process to assess draft guideline recommendations. This feedback will then be given back to developers to consider when finalizing the recommendations. As guideline characteristics are best assessed by end-users, the objectives of the current study were to explore how family physicians perceive guideline implementability, and to determine what components should comprise the final GUIDE-IT prototype.

**Methods:**

We conducted a qualitative study with family physicians inToronto, Ontario. Two experienced investigators conducted one-hour interviews with family physicians using a semi-structured interview guide to 1) elicit feedback on perceptions on guideline implementability; 2) to generate a discussion in response to three draft recommendations; and 3) to provide feedback on the conceptual GUIDE-IT. Sessions were audio taped and transcribed verbatim. Data collection and analysis were guided by content analyses.

**Results:**

20 family physicians participated. They perceived guideline uptake according to facilitators and barriers across 6 categories of guideline implementability (format, content, language, usability, development, and the practice environment). Participants’ feedback on 3 draft guideline recommendations were grouped according to guideline perception, cognition, and agreement. When asked to comment on GUIDE-IT, most respondents believed that the tool would be useful, but urged to involve “regular” or community family physicians in the process, and suggested that an online system would be the most efficient way to deliver it.

**Conclusions:**

Our study identified facilitators and barriers of guideline implementability from the perspective of community and academic family physicians that will be used to build our GUIDE-IT prototype. Our findings build on current knowledge by showing that family physicians perceive guideline uptake mostly according to factors that are in the control of guideline developers.

## Background

Clinical practice guidelines are considered an important knowledge translation tool to inform clinical practice for family physicians, yet their potential has not been realized due to inconsistent adoption in clinical practice [1-3]. Guidelines can be confusing and difficult to use (and even contradictory), and their implementation and uptake remain variable [4,5]. The research community classifies inquiry about poor guideline adoption into strategies that focus on the practice or environment (extrinsic approaches) or the guideline itself (intrinsic approaches) to improve uptake. Extrinsic strategies are important since gaps in uptake and use continue to exist even if guidelines are well developed and implementable. However these strategies have had modest impact on quality of care with highly variable costs [6]. We believe that optimising the intrinsic characteristics of guidelines (e.g., wording and format) that are associated with uptake may have greater potential to improve uptake at minimal cost, be easier to implement, and may be broadly applicable.

*Implementability* refers to a set of characteristics that “predict ease of (and obstacles to) guideline implementation” [7], whereas *Implementation* refers to that part of the guideline lifecycle in which systems are introduced to influence clinicians' behavior toward guideline adherence [8].There has been much discussion around improving the rigor of guidelines [9], but few tools consider implementation issues during guideline development with the expectation of improving guideline uptake. Existing tools include GLIA [7], which informs developers about potential problems with implementation, but it is time consuming to use and difficult to incorporate into the development process, and not designed to assess the implementability of the whole guideline [10]; AGREE [11] can assess the methodological quality of guidelines but provides only peripheral guidance on implementability; and ADAPTE [12] can be used to adapt existing guidelines into other settings. However, these tools are not based on a broad literature search, and most target methodological and reporting concerns. Currently, there is no resource that takes a comprehensive view of all factors relevant to guideline implementability. Guideline developers need a usable, complementary tool to improve the implementability of guideline recommendations. We developed a conceptual tool with the aid of a general model for planned action (i.e., the knowledge-to action [KTA] framework) [13]. The tool development began with an exhaustive review using Realist Review methodology to better understand the intrinsic characteristics of guidelines that impact on their implementability [14]. Findings of this study are reported in another paper ([15], Kastner M, Hayden L, Makarski J, et al. Understanding the implementability of clinical practice guidelines: A Realist Review. Submitted), but briefly, it identified core implementability dimensions known to influence uptake: *Stakeholder involvement*, *Evidence synthesis*, *Considered judgment*, *Feasibility*, *Message*, and *Format*. The “conceptual” tool, which we call the Guideline Implementability Tool (GUIDE-IT), was designed according to these Realist Review findings in addition to consultation with experts in guideline development. We conceptualized GUIDE-IT as a 3-step process:

1) A strategy to recruit and involve family physicians (i.e., the typical end-users of clinical practice guidelines) in the guideline development process;

2) A process to evaluate the implementability of draft recommendations by family physicians recruited in step 1 using an objective assessment checklist (online or paper-based platform); and

3) Provision of family physician assessments to guideline developers (who will also evaluate recommendations) to take into consideration when finalizing recommendations.

Once we developed the conceptual GUIDE-IT tool, the next steps in the tool development process (according to our KTA framework) were to better understand the barriers to using the knowledge gleaned from our Realist Review and to transform our conceptual tool design into a functional prototype. To do this, we wanted to expand our understanding of guideline implementability from the perspective of family physicians, which represent a large proportion of guideline end-users. As guideline characteristics are best assessed by end-users, we wanted to explore how family physicians perceive this concept in the context of their practice and to determine how they respond to our conceptual GUIDE-IT tool. Thus, the specific objectives of this study were to explore 1) what guideline end-users (family physicians) perceive as important in guidelines, guideline development and uptake (i.e., what they consider facilitators and barriers); 2) to determine how family physicians perceive a set of draft guideline recommendations; and 3) to determine what components should comprise the final GUIDE-IT prototype.

## Methods

We conducted a qualitative study of interviews with community and academic family physicians. Our study was planned and executed in accordance with RATS guidelines (http://www.biomedcentral.com/authors/rats). We used a mixed-methods sampling strategy in an attempt to recruit a wider scope of family practice. We purposively recruited family physicians from the St. Michael’s Hospital (SMH) family practice unit in Toronto, Ontario (N = 43). However, we needed to extend our recruitment beyond our research institute family practice unit (which predominantly comprise academic family physicians) to gain a realistic understanding of guideline implementability. To identify a broader spectrum of family practice perspectives (including physicians in academic and community family practice settings), we also randomly selected physicians from the College of Physicians and Surgeons of Ontario (CPSO) database in the greater Toronto area (N = 962). We performed this mixed recruitment strategy simultaneously until we reached data saturation. Sampling was stratified by type of physician (academic or community) in an attempt to maximize the potential for recruiting an equal representation of each. Based on our previous experience conducting qualitative interviews, we anticipated having to interview 10-15 physicians to achieve data saturation. Once providing informed consent, all participants were assigned a unique identification number to ensure the anonymity of their responses. The study was approved by the St. Michael’s Hospital Research Ethics Board.

### Interview sessions

Two experienced investigators conducted one-hour interviews with family physicians using a semi-structured interview guide (Additional file 1) with the following 3 objectives that were planned in sequence:

In Objective 1, we wanted to elicit feedback on participants’ perceptions of guidelines, and their views on the facilitators and barriers to using them.

In Objective 1, we wanted to elicit feedback on participants’ perceptions of guidelines, and their views on the facilitators and barriers to using them.

In Objective 2, the aim was to generate a discussion in response to 3 draft recommendations provided by a guideline development group (Additional file 2). Participants were shown the 3 draft recommendations one at a time, and asked to respond to each using the following questions from the interview guide:

1. *What do you think the recommendation is saying? Do you understand it? If no, what aspects do you find difficult?*

2. *What would you do with this recommendation in practice?*

3. *Would you change this recommendation? Why/why not? How would you change it?*

**Figure 1:**
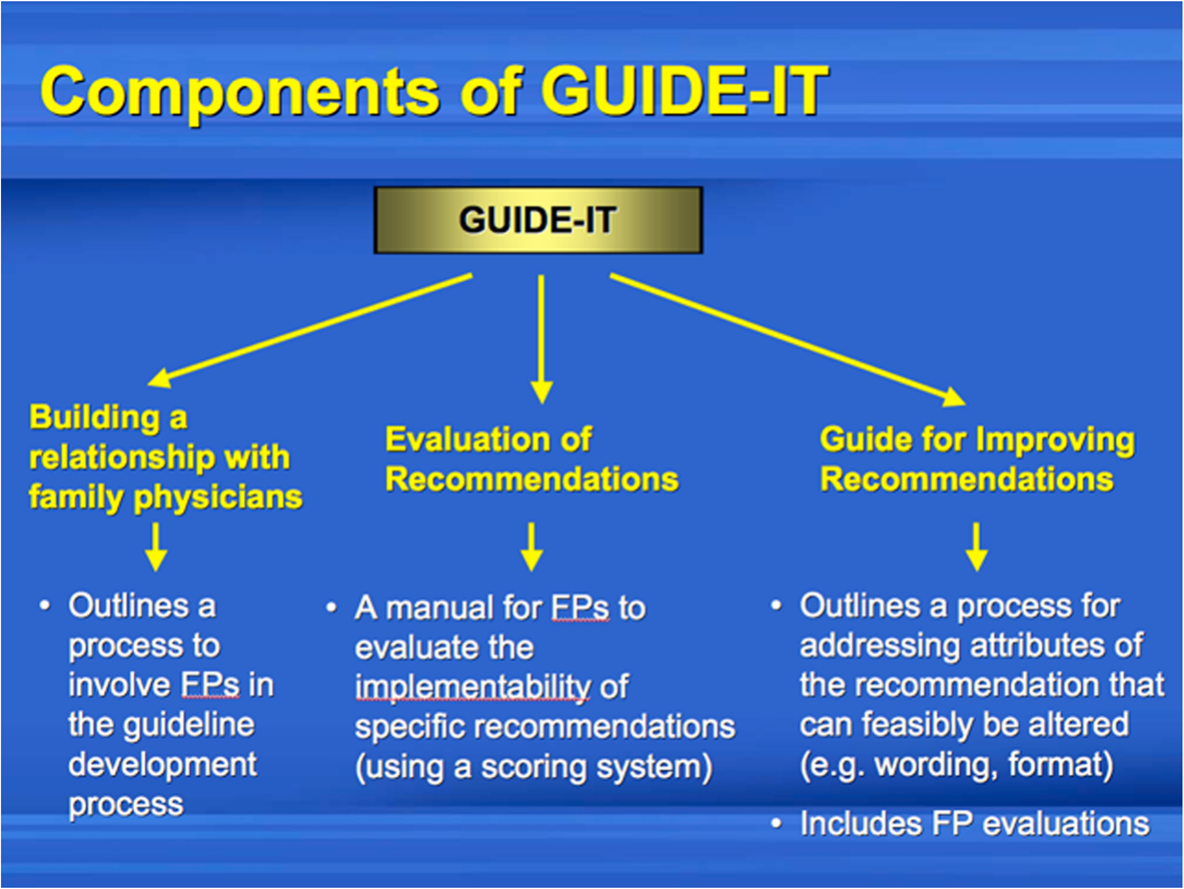
Conceptual design of the guideline implementability tool (GUIDE-IT) that was presented for family physicians during the interviews (Objective 3).

In Objective 3, we wanted to elicit feedback on our conceptual guideline implementability tool (GUIDE-IT), including the generation of ideas and suggestions about optimal features that might enhance guideline uptake. The tool was developed based on the expert input of guideline developers and findings of our realist review investigating characteristics of guidelines that facilitate their uptake [14]. The qualitative interviews in the current study represent guideline end-user perspectives, which will contribute to other stakeholder perspectives in the development of the final prototype. Figure 1 shows the conceptual GUIDE-IT design that was presented to participants during the last part of the interview session.

### Data collection and analysis

Sessions were audio taped and transcribed verbatim. Data collection and qualitative content analysis were guided by grounded theory methodology [16]. Four investigators independently developed a coding scheme by identifying, and labelling the primary patterns in the content using NVivo [8.0]. For Objectives 1-3, we used the constant comparative method to group codes into categories (where each category was considered as a unit of analysis). For example, codes for the “clarity”, “specificity”, “actionability” and “sensibility” of guidelines were grouped into a category called “Language”. We also interrogated the inter-relationship of categories (axial coding). For example, we had group discussions to determine whether two broad categories, “Content” and “Language” of guidelines, should be combined or represented as two separate categories. After coding the data and reviewing the content related to participants’ feedback on the three draft guideline recommendations (Objective 2), we grouped participant responses according to the theory of information architecture [17,18]. This theory states that people perceive information before processing it and that format/layout can influence a person’s ability to process information at both the perception and cognition level. We also recognized that beyond cognitive understanding, our informants spoke of a third level, which was assessment. Once they understood the basic message, they assessed whether it was logical, correlated with prior experience, and whether they could see themselves following the recommendation in practice. As such, we conducted axial coding for this level of analysis. To increase our validity, we also performed data triangulation, by seeking input from other researchers and experts in guideline development on our themes, categories and analysis.

## Results

Of 1005 family physicians who were invited, 22 agreed to participate (N = 14 from the CPSO; N = 8 from SMH, of whom 5 were also in the CPSO database). Of these, 2 physicians did not attend, so we conducted interviews with a total of 20 family physicians (10 one-on-one and 4 group sessions). Table 1 shows the characteristics of participants. Most were community family physicians (70%) practicing for at least 16 years (60%) and younger than 55 years of age (65%) (Table 1). Results are described below according to our 3 major objectives.

**Table 1:** Characteristics of family physicians (N = 20)

**Characteristic**	**N (%)**
**Gender**	
Women	11 (55)
	Men	9 (45)
**Age range (years)**		
25-35	5 (25)	
36-45	5 (25)	
46-55	3 (15)	
56-65	6 (30)	
>65	1 (5)	
**Type of practice**		
Community	14 (70)	
Academic	6 (30)	
**Years in practice (years)**		
< 5	2 (10)	
5-10	4 (20)	
11-15	2 (10)	
16-25	6 (30)	
>25	6 (30)	

### Objective 1: Perception of the facilitators and barriers to guideline use

**Figure 2:**
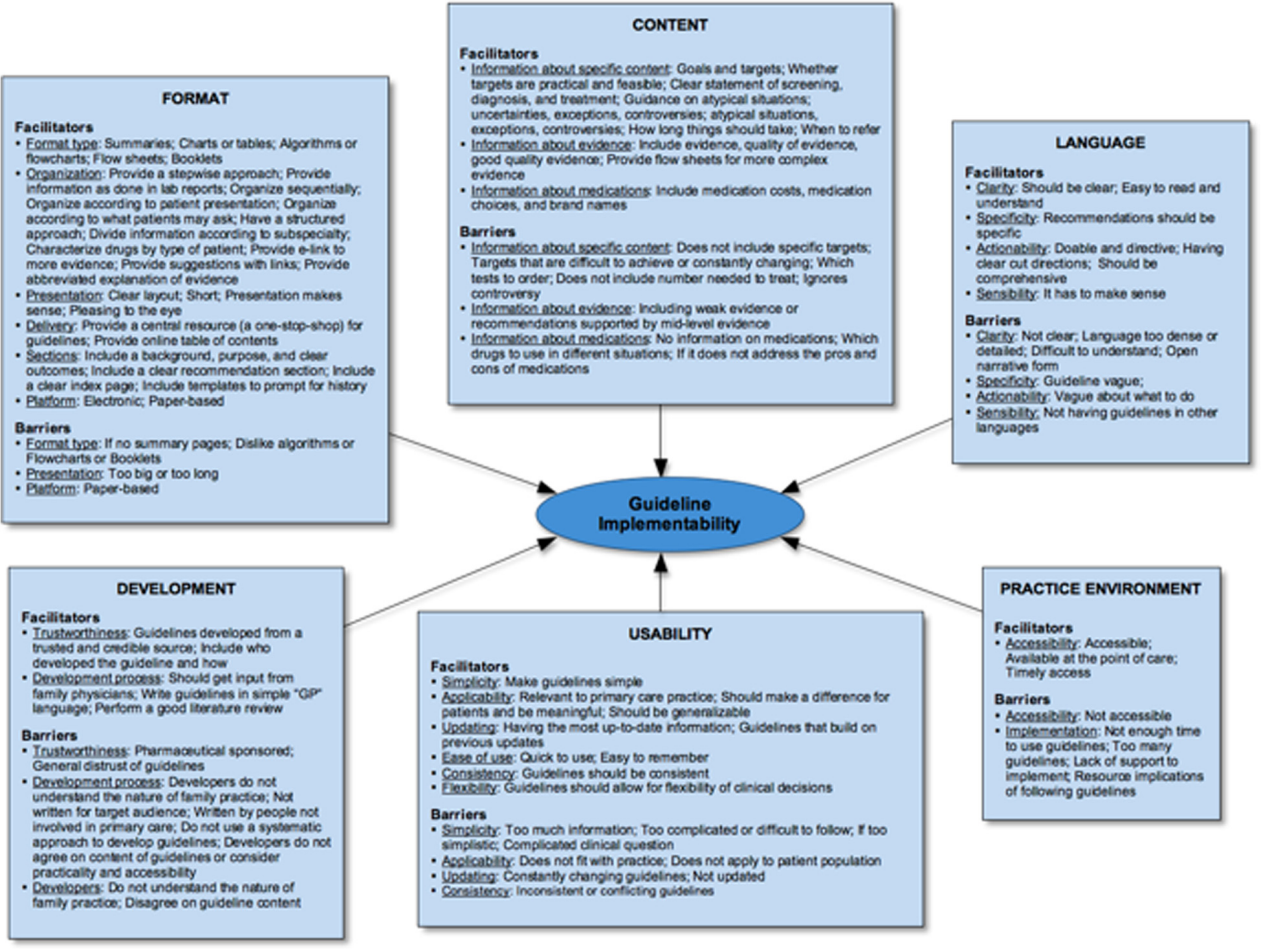
Categories of guideline implementabiliy as perceived by family physicians.

Family physicians perceived guideline uptake according to facilitator and barrier factors spread across 6 categories of guideline implementability: Format, Content, Language, Usability, Development, and the Practice environment (Figure 2).

Format (Table 2): The majority of family physicians identified a preference for the representation of guidelines as summaries, charts or tables. Suggestions related to the organization of guidelines included a stepwise approach, providing information similar to how it is laid out in lab reports, and according to what patients may ask. In terms of presentation, physicians want a clear layout that makes sense and is “pleasing to the eye”. Some suggested that the provision of a “central resource (a one-stop-shop)” for guidelines would be a good delivery system. Sections identified by participants as important to include in guidelines were a background, purpose, outcomes, a clear recommendation section, and templates to prompt for history. Guidelines described as too big or too long were perceived as the major barriers to uptake.

**Table 2:** Facilitators and barriers of guideline implementability as perceived by Family Physicians: FORMAT

**Facilitators *(Number of respondents)***		**Utterance example**	**Barriers *(Number of respondents)***	**Utterance example**
**Format type**				
	**Summaries (N = 8)**	*● It would be helpful to have a quick summary because the guideline will be read at one point but not while the patient is there. {I-8}*	**If no summary pages (N = 1)**	*● If there was no summary pages provided {FG4-P2}*
	**Charts or tables (N = 5)**	*● Tabular form would be the most useful because it is very clear. {I-3}*	-	-
		*● I appreciate having tables, charts, flow charts - I usually refer to these {I-4}*		
	**Algorithms or flowcharts (N = 3)**	*● If I can't discern where the guideline's direction is then that's how you know that sometimes algorithms are really much more helpful because we only have maybe 10, 15 minutes per patient. {FG1-P2}*	**Algorithms/Flowcharts (N = 3)**	*● Flowcharts are too complex and confusing {FG4-P2}*
		*● Clear decision trees in terms of how to go through management decision making processes. {I-1}*		*● I am not crazy about algorithms – it takes a while to get through it; Even if the guideline is no longer than 15 pages, if there are a lot of algorithms and a lot of text, it sort of goes out of my mind. – I don’t like them and find the print too small {I-3}*
	**Flow sheets (N = 3)**	*● A great tool is flow sheets that can be incorporated into practice and be used to track patients' progress according to guideline recommendations, it’s easy to use and follow {I-1}*	-	*-*
	**Books or booklets (N = 2)**	*● The guidelines I use more frequently are in a booklet form with an index so you can look up what you want {I-3}*	**Booklets (N = 1)**	*● I get turned off very quickly if I get booklets {FG1-P2}’*
		*● If you provide booklets, have it colour coordinated {FG2-P2}*		
	**Laminated cards (N = 1)**	*● Guidelines boiled down to a one page laminated card that is easy to use (e.g., diabetes flow sheet published by the ministry) {I-1}*	-	*-*
	**Checkboxes (N = 1)**	*● Checkboxes are wonderful - having clear check boxes or something to say that the test was completed and indicative of the diagnosis; have some of the checklists compatible to billing purposes {I-2}*	-	*-*
	**Desk calendar (N = 1)**	*● A desk calendar format (e.g., diabetes guidelines) is user-friendly, readable but evidence-based and accessible as it can go on a desk or shelf as a visual reminder of the guideline. {FG1-P3}*	-	*-*
**Organization (N = 16)**				
	**Provide a stepwise approach (N = 2)**	*● Guidelines are useful if they provide a staged, progressive approach so if you are stuck you can keep going through stages {FG3-P1}*	-	-
	**Provide information as done in lab reports (N = 2)**	*● I wouldn't be able to recall the numbers and ranges from cholesterol and diabetes guideline, but they are in every lab report so I can't help but use them and discuss with patients - why can't guidelines do the same? {FG2-P2}*	-	-
	**Organize sequentially (N = 1)**	*● Guideline recommendations should be sequential - it should say why we're choosing this particular option (we can go this way or that way) {I-7}*	-	-
	**Organize according to patient presentation (N = 1)**	*● Having materials well organized into blocks of presentation: presenting complaint, family history, reason for problem {I-2}*	*-*	*-*
	**Organize according to what patients may ask (N = 1)**	*● Methods of presentation that geared toward questions that patients are likely to ask {I-1}*	-	-
	**Have a structured approach (N = 1)**	*● Having a structured approach so that no detail is forgotten. {I-2}*	-	-
	**Divide information according to sub-specialty (N = 1)**	*● I can't keep track of all the names of the studies - ones from cardiovascular, osteoporosis, diabetes. So, they could be divided up according to sub-specialty {FG2-P1}*	-	-
	**Characterize drugs by type of patient (N = 1)**	*● I like the ones that integrate into the way do things anyway (which drug to choose first, like in hypertension). It lays it out clearly and characterize the type of patient that would do better with this vs that drug {I-8}*	*-*	*-*
	**Provide e-link to more evidence (N = 1)**	*● Provide an e-link to the evidence so that doc can verify appropriateness of recommendation for their patient; include outcomes with links to evidence supporting recommendations; for reassurance of recommendation and for use to educate patients {FG1-P2}*	-	-
	**Provide suggestions with links (N = 1)**	*● Provide suggestions with links so it can be drilled down {FG1-P1}*	-	-
	**Provide abbreviated explanation of evidence (N = 1)**	*● Could provide an addendum of a very simplified version of the more popular studies - an abbreviated explanation of the study and its findings {FG2-P1}*	*-*	*-*
**Presentation (N = 13)**				
	**Clear layout (N = 2)**	*● Clearly and accessibly laid out for management {I-1}*	*-*	*-*
	**Short (N = 2)**	*Short guidelines (3 pages) are easy to read, understand and implement (the way old diabetes guidelines used to be) {I-6}*	**Too big/too long (N = 7)**	*● It’s frustrating when they are super big {FG4-P1}*
				*● If it has 4 or more pages, I don’t even look at it {FG2-P2}*
	**Pleasing to the eye (N = 1)**	*● Methods of presentation that are pleasing to the eye {I-1}*	-	*-*
	**Presentation makes sense (N = 1)**	*● Methods of presentation that make sense {I-1}*	-	*-*
**Delivery (N = 4)**				
	**Provide a central resource (a one-stop-shop) for guidelines (N = 3)**	*● The ideal guideline would be a one-stop-shop like "Up-to-date" which I use because I know I just go there and get the answer.; I would love to have a central resource which contains all of the most trusted and strongest guidelines, where I can go, search the information I want and have the answer pop up {I-1}*	***-***	*-*
	**Provide online table of contents (N = 1)**	*● Include an online table of contents where you can quickly get to the info and click the section you are looking for {I-5}*	***-***	*-*
**Sections (N = 6)**				
	**Include a background (N = 1)**	*● Provide background {I-1}*	**-**	*-*
	**Include purpose of the guideline (N = 1)**	*● Simple articulation of the purpose of the guideline is extremely important {I-2}*	**-**	*-*
	**Include clear outcomes (N = 1)**	*● Include clear outcomes {FG1-P2}*	**-**	*-*
	**Provide a clear recommendation section (N = 1)**	*● They have a very clear recommendation section at the end of any article that you can always flip to and then go back, they will also have a lot of the supporting evidence there so it kind of provides a nice balance in that way {I-1}*		
	**Provide a clear index page (N = 1)**	*● Provide a clear index page {I-9}*		
	**Include templates to prompt for history (N = 1)**	*● Providing a template of information to prompt for family history or date of last check up or other details and comprehensive review of the problem would facilitate the use of guidelines and make more time for family doc {I-2}*	**-**	*-*
**Platform (N = 4)**				
	**Electronic (N = 3)**	*● Having it online at some free location where you don't have to login would be good {I-5}*	***-***	*-*
	**Paper-based (N = 1)**	*● A useful guideline is one that is available at the POC - so that comes in either a convenient hard copy document (e.g., CHEP, which has the summary packet on hypertension guidelines) that you can search very quickly as opposed to the diabetes guidelines which are several hundred pages {I-5}*	**Paper-based (N = 1)**	*● I don’t want paper-based {FG2-P2}*

Content (Table 3): Physicians identified the importance of including information about goals and targets (and whether these are practical and feasible), medication costs and choices, and to provide guidance on atypical situations, exceptions, controversies and uncertainties. Targets that do not fit with practice, do not apply to the patient population, and are difficult to achieve or constantly changing were identified as barriers. Many physicians thought it was important to include evidence and its quality in guidelines, but some believed that only recommendations with good quality of evidence should be included, while recommendations with weak or mid-level evidence should not.

**Table 3:** Facilitators and barriers of guideline implementability as perceived by Family Physicians: CONTENT

**Facilitators *(Number of respondents)***		**Utterance example**	**Barriers *(Number of respondents)***	**Utterance example**
**Specific content to include (N = 17)**				
	**Goals and targets (N = 2)**	*● Provide easily understandable goals and targets that can be applied in practice. {I-1}*	**Does not include specific targets (N = 1)**	*● Some guidelines like Diabetes are hard to follow because they include sections at the end with special groups. It’s important that these subgroups [aboriginal] are mentioned because they are higher risk group and you have to keep a closer eye on them for complications but the recommendations become more defuse or nebulous or vague that obviously come out of some evidence base but don’t provide specific targets to follow and are not useful for every day frontline practice situations {I-1}*
		*● Like to see a clear statement of the targets aimed for {FG4-P2}*		
	**Whether targets are practical and feasible (N = 1)**	*● Whether guidelines are practical, feasible are the most important in terms of the numbers that should be targeted (for example in hypertension guidelines) in terms of the first, second, third-line medications {I-4}*	**Targets that are difficult to achieve (N = 2)**	*● In hypertension guidelines it’s very hard to achieve the targets that they recommend {I-4}*
			**Targets constantly changing (N = 1)**	*● Sometimes targets [such as in Cholesterol guidelines] are unattainable because they are always changing and difficult to achieve in the real world {FG3-P2}*
	**Clear statement of screening, diagnosis and treatment (N = 1)**	*● Like to see a clear statement of the screening and diagnosis or management, and the targets aimed for {FG4-P2}*	**Which tests to order (N = 1)**	*● There is a point of frustration for me sometimes, like what tests to order [Right] you know, like for TSH {FG4-P1}*
	**Guidance on atypical situations (N = 1)**	*● Guidelines should provide guidance on what is atypical - to know the procedure or how to investigate the red flags in a timely manner; to know what to do and is appropriate for things that are not as common like retinal detachment - to know the red flags and what to look for and what procedure to investigate in a timely manner {I-8}*	**Does not include number needed to treat (N = 1)**	*● The thing I always look at is, is the sort of how many people needed to treat you know, so if I have to put 1000 of my patients on beta blockers to save one MI you know, that's not going to be implemented or the guideline I am going to get very excited about. So the number needed to treat, are the things I think are not available in guidelines and should be, because that's the evidence. {FG1-P3}*
	**Guidance on uncertainties (N = 1)**	*● It's good that Hypertension guidelines address uncertainties so you know what to do for high risk, intermediate and low risk patients (e.g., controversies around the secondary markers besides LDL and HDL). {I-6}*	**-**	*-*
	**Guidance on exceptions (N = 1)**	*● Include exceptions and how to deal with them {I-1}*	**-**	*-*
	**Controversies (N = 1)**	*● In some cases, you would want more information. They have to show the controversy and they've got to show how they've looked at both sides {I-6}*	**Ignores controversy (N = 1)**	*● It isn't helpful if a guideline doesn’t address areas of controversy or ignores it {I-6}*
	**How long things should take (N = 1)**	*● In some investigations where timeline is an issue, it would be helpful if guidelines included information on how long it should take or don't leave it more than this long or investigate this and then this or do all {I-8}*	**-**	*-*
	**When to refer (N = 1)**	*● It would be good to know when to refer. I sometimes refer and then the specialists comes back to ask why I referred {FG2-P2}*	**-**	*-*
**Information about evidence (N = 16)**				
	**Include evidence (N = 5)**	*● Provide evidence and its quality {I-1}*	**Including weak evidence (N = 3)**	*● Grade D evidence should not be considered or included in guidelines {FG1-P3}*
		*Provide the evidence behind recommendations {FG1-P2}*		
	**Include information on quality of evidence (N = 6)**	*● Guidelines should provide the strength and quality of evidence with the statement {FG1-P1}*	**Recommendations supported by mid-level evidence (N = 1)**	*● The most difficult is to follow a guideline with mid-level type of evidence where I’m not quite sure which direction to follow – If its B, C or D, it is against consensus which is what makes it much more difficult – I question using that particular guideline and makes it difficult for me to implement that into my practice {FG1-P2}*
		*● If it’s Grade A evidence, then I feel much more comfortable using that {FG1-P2}*		
	**Provide flow sheets (N = 1)**	*● For more complex evidence, provide flow sheets {FG1-P1}*		
**Information about medications (N = 15)**				
	**Include medication costs (N = 6)**	*● Specialists will say that patients with hypertension are not being treated adequately and family physicians are not doing a good job but sometimes it’s difficult to follow guidelines because of cost of medication and so we can’t do exactly what the guidelines say {FG3-P3}*	**No information on medications (N = 1)**	*● Many times, I get a report back that is totally useless because it’s not telling me whether or not the drug my patient is taking is effective or not, that is, it’s not clear whether it’s simply not reported in the guideline because the guideline committee decided to leave out this information {I-10}*
	**Include medication choices (N = 5)**	*● Guidelines should point in the right direction if the first or second-line treatments aren't going to work for whatever reason. I like how the hypertension guideline help you choose the right drug - the ones that integrate into the way do things anyway (which drug to choose first). It lays it out clearly and characterize the type of patient that would do better with this drug vs another - particularly in my practice where I have a multicultural practice because certain populations are different and you want to know that {I-8}*	**Does not address the pros and cons of medications (N = 1)**	*● Guidelines don’t strongly address the pros and cons of medications (e.g., bisphosphonates for osteoporosis) {I-6}*
	**Include brand names (N = 1)**	*● Guidelines could provide the brand names in brackets for the common medications. {FG2-P1}*		

Language (Table 4): Many family physicians perceive guidelines as using language that is too dense or too detailed. They want guidelines to be clear, easy to read and understand; and are specific, actionable and make sense.

**Table 4:** Facilitators and barriers of guideline implementability as perceived by Family Physicians: LANGUAGE

**Facilitators *(Number of respondents)***		**Utterance example**	**Barriers *(Number of respondents)***	**Utterance example**
**Clarity (N = 18)**				
**Clear (N = 6)**		*● Guidelines need to be clear {I-6}*	**Language too dense or detailed (N = 8)**	*● When it comes to wording of recommendations, if there is too much information in a single recommendation, the reader gets lost in it. {I-5}*
**Easy to read and understand (N = 2)**		*● Guidelines need to be easy to read and understand {I-6}*	**Difficult to understand (N = 1)**	*● If recommendation is hard to understand, I just glaze over it {I-2}*
**Specificity (N = 11)**				
**Specific (N = 7)**		*● Guidelines need to be as specific as possible to the question {I-2}*	**Guideline vague (N = 4)**	*● If the guideline is vague, if for example 2 medications fits all approaches then I find that not very useful {I-3}*
**Actionability (N = 7)**				
**Doable and directive (N = 3)**		*● It should guide the questions that you are asking and get you to the decision making point in the guideline; Guidelines should direct me through the end stages of management and diagnostic processes so it gets to the bottom line recommendations {I-1}*	**Vague about what to do (N = 2)**	*● Often the actual recommendation does not answer the question – it may give you a direction but you may need to dig deeper to find out how to actually carry out the recommendation {I-5}*
	**Having clear cut instructions (N = 1)**	*● If you pushed for time or you have extenuating circumstances or a, a visit includes parents or other informants that may have different ideas you eliminate the distractibility of not completing the information you do need by having clear cut instructions as to what should be included. {I-2}*		
	**Should be comprehensive (N = 1)**	*● Something that's comprehensive so that if you’re pushed for time or you have extenuating circumstances or a visit includes parents or other informants that may have different ideas you eliminate the distractibility and have clear cut instructions as to what should be included {I-2}.*		
**Sensibility (N = 3)**				
	**It has to make sense (N = 2)**	*● It has to make sense, whatever the new thing is. So often the guideline comes out and I will often sit back and say, I don't think this really makes sense, and it doesn't resonate with me, like HRT. {I-6}*	**Not having guidelines in other languages (N = 1)**	*● One barrier may be language, not having things available in the language of the physician or patient. Some of the materials need to be filled in by the patients as well and if they don't understand the date of last pap smear was, then the information is going to have limited use {I-2}*
		*● Guidelines need to be intuitive and logical {FG4-P2}.*		

Usability (Table 5): Most family physicians perceive guidelines as having too much information, and generally too difficult to follow. Guidelines that do not fit with their practice or apply to their patient population, and are inconsistent or conflicting were also major perceived barriers to use. Family physicians want guidelines to be relevant to primary care practice, make a difference for patients and be meaningful. They also want guidelines that are quick to use and easy to remember, but allow for enough flexibility to apply their own judgment to make practice decisions. Having the most up-to-date guideline information or those that are built on previous updates was also considered a facilitator, but only if guidelines are not constantly changing.

**Table 5:** Facilitators and barriers of guideline implementability as perceived by Family Physicians: USABILIY

**Facilitators *(Number of respondents)***	**Utterance example**	**Barriers *(Number of respondents)***	**Utterance example**
**Simplicity (N = 14)**			
**Simple (N = 4)**	*● Keep it simple and straightforward {FG1-P3}*	**Too much information (N = 3)**	*● It’s not helpful if the guideline requires some reading, and people have varying levels of patience and time to read around the recommendations to find out how they are supposed to implement the recommendation {I-5}*
	*● Guidelines have to be simple and straightforward, if it’s complicated I’m not going to remember it anyway and won’t look at it {FG2-P2}*		
		**Too complicated or difficult to follow (N = 4)**	*● Some guidelines are very complex (i.e., the ones with 30 recommendations) and written above the practice level, so will not go back to it because its overwhelming and a bombardment of information - no time to fit into brain. There should be 5 of the most important things that should be given to family physicians (by specialists) to remember {I-1}*
		**If too simplistic (N = 2)**	*● Guidelines are meant to simplify but sometimes they become too simplistic {FG3-P3}*
		**Complicated clinical questions (N = 1)**	*● We struggle with the complex management questions (e.g., if there is 4 second degree relatives but no first degree relative when considering mammography at age 40 instead of age 50) {I-10}*
**Applicability (N = 11)**			
**Relevant to primary care practice (N = 1)**	*● Answers questions that are relevant to primary care and thus clinically useful {I-10}*	**Does not fit with practice (N = 5)**	*● What they tell you to do and what you actually do in your practice never seem to match up {I-8}*
**Should make a difference for patients (N = 1)**	*● So, I think one of the things that I would want to see is if it makes a difference to my patient you know, just having a lower level or having everybody with a hemoglobin A1C of whatever, it doesn't always translate into healthier patients {FG1-P3}*	**Does not apply to patient population (N = 3)**	*● Applicability to primary care population is a barrier; I think it's also that specialists see a particular population and they are coming from a particular viewpoint. But then, as a primary care physician, your population is going to be a little bit different, so I think that's where the challenge lies {I-9}*
**Should be meaningful (N = 1)**	*● The number needed to treat has to be meaningful to make it implementable (putting 1000 people on beta blockers to save 1 MI is not implementable) {FG1-P3}*	**-**	*-*
**Updating (N = 8)**			
**Having the most up-to-date information (N = 1)**	*● One quality that would influence my decision making is guidelines that are current and have the most up-to-date information {I-2}*	**Constantly changing guidelines (N = 4)**	*● Changing/update of evidence forces changes to routine practice that is difficult to explain to patients {FG2-P1}*
**Guidelines that build on previous updates (N = 1)**	*● Guidelines that are helpful are those that build on previous updates (e.g., blood pressure and hypertension guidelines), the ones that build on what you already know {I-9}*	**Guidelines that are not updated (N = 2)**	*● There are also recommendations which don’t seem to fit with what I know of the latest evidence, where I start wondering, okay just how up to date is this thing, I mean that’s another barrier to it. {I-1}*
**Ease of use (N = 6)**			
**Quick to use (N = 4)**	*● Guidelines should be quick to use because family physicians' approach to seeing patients is different than specialists - less time to reflect on how to manage {I-3}*	**-**	*-*
**Easy to remember (N = 2)**	*● Something that sticks in my mind and can be used on a day-to-day basis {I-1}*	**-**	*-*
**Consistency (N = 5)**			
**Consistent (N = 1)**	*● I mean we forget about the rest of the world {Laughter} I mean you have guidelines from every country but at least in Canada they're getting some consistency in terms of you know, the big diseases which is nice {FG4-P2}*	**Inconsistent or conflicting (N = 4)**	*● Recommendations that are not firm and sort of all over the place (e.g., one guideline states to exercise 30 minutes/day while another states 5 minutes/day); this was a big issue when hypertension and cholesterol guidelines did not sync with diabetes. {I-1}*
**Flexibility (N = 3)**			
**Flexible (N = 3)**	*● Guidelines have to be flexible; Sometimes there is no flexibility when they say you can do this and then next step is first line, second like – this should be fairly straightforward but in reality it’s not always easy to do that {FG1-P3}*	**-**	-
	*● Guidelines should allow you to be creative and let you think outside of the box in case something doesn't fit in smoothly {I-2}*		

Development (Table 6): Our participants believed that guidelines should be developed by a trusted and credible source with input from family physicians. Some believed that the specialists who develop guidelines do not understand the nature of family practice and as such are not the appropriate persons to write them. A few family physicians expressed a lack of trust in guidelines either because they questioned the “solidity of recommendations” or because they were industry sponsored.

**Table 6:** Facilitators and barriers of guideline implementability as perceived by Family Physicians: DEVELOPMENT

**Top facilitators *(Number of utterances)***		**Utterance example**	**Top barriers *(Number of utterances)***	**Utterance example**
**Trustworthinesss (N = 13)**				
	**Guidelines from a trusted and credible source (N = 5)**	*● I need to see that the guideline has been endorsed by credible sources [FG1-P1 I trust guidelines developed by a central committee of experts that are recognized from across the country such as diabetes or hypertension – when I trust a guideline, it is easy to understand and commit to memory and refer back to it over and over again {I-1}*	**Pharmaceutical sponsored (N = 4)**	*● I will flip very quickly over to who sponsored it, and if there is a pharmaceutical company on it, then I tend to take it with a grain of salt. So, those are also called guidelines and sometimes it's hard to distinguish {I-6}*
	**Include who developed the guideline and how (N = 1)**	*● It’s important to know who developed the guideline and how they developed it. {I-6}*	**General distrust of guidelines (N = 3)**	*● Just because something is a guideline, I don’t necessarily trust that it is not conflicted, so that’s a concern for me {I-1}*
**Development process (N = 12)**				
	**Developers should get input from family physicians (N = 1)**	*● Specialists writing the guidelines should probably get input from family physicians - for hypertension, it says use this medication because it's wonderful, but when you are facing the patient, they tell you that they can't afford it {FG4-P1}*	**Developers do not understand the nature of family practice (N = 2)**	*● They [guideline developers] should realize that an appointment is 10 minutes and you need to deal with an issue, prescribe the medication, and talk about the risks and side effects in that 10-minutes, and if you can't deal with all the issues in 10 minutes then the guideline is too cumbersome {FG4-P2}*
	**Developers should write guidelines in simple “GP” language (N = 1)**	*● Put guidelines in simple “GP” language {FG2-P1}*	**Not written for target audience (N = 3)**	*● So I don't think they are clear as to exactly who they were writing for: for doctors in general or just for family doctors {FG1-P1}*
			**Written by people not involved in primary care (N = 1)**	*● It’s written by people who are likely not involved in primary care. We need something that you can very quickly skim through, and they are not always created that way {FG1-P1}*
	**Developers should perform a good literature review (N = 1)**	*● You want to feel that guideline developers have looked at all the information and did a good literature review. {I-6}*	**Developers do not use a systematic approach to develop guidelines (N = 1)**	*● I don't think guidelines are developed using a systematic approach, and it's a slow process {FG4-P1}*
			**Developers do not agree on content of guidelines (N = 1)**	*● So, among specialists, I don’t think they agree on the guidelines anyway. Some specialists have different opinions than those in guidelines {FG2-P2}*
			**Developers do not consider practicality and accessibility (N = 1)**	*● Guideline developers focus is the content of the recommendations not the accessibility or practicality of it, so they see it from a different perspective. {FG4-P2}*

Practice environment (Table 7): Participants wanted guidelines to be accessible and available at the point of care, but many have difficulty finding them or simply do not know that they are available. Almost half of participants indicated that lack of time was a major barrier to implementation of guidelines. Other perceived barriers were too many guidelines and a lack of support to implement them, and that available resources are not taken into consideration when designing guidelines to be able to appropriately implement them.

**Table 7:** Facilitators and barriers of guideline implementability as perceived by Family Physicians: PRACTICE ENVIRONMENT

**Top facilitators *(Number of utterances)***		**Utterance example**	**Top barriers *(Number of utterances)***	**Utterance example**
**Accessibility (N = 12)**				
	**Should be accessible (N = 3)**	*● Accessibility of the information is a factor that will impact on implementability {I-9}*	**Not accessible (N = 6)**	*● Guidelines are not accessible {FG1-P3}*
				*● If I have to look for it, it will not happen as easily {FG2-P2}*
				*● One thing I find difficult about using different guidelines is just finding them when I need them {Laughter} {I-8}*
		*● It has to be something that can be handy and accessible {I-5}*		
				*● Guideline developers focus is the content of the recommendations not the accessibility or practicality of it, so they see it from a different perspective {FG4-P2}*
	**Available at the point of care (POC) (N = 2)**	*● A useful guideline is one that is available at the POC - so that comes in either a convenient hard copy document that you can search very quickly as opposed to the diabetes guidelines which are several hundred pages. Osteoporosis has a little one-pager that was sent out with the journal {I-5}*		
				*● One of the barriers is not realizing that the guidelines are available {I-2}*
				*● One of the big problems in terms of accessibility with guidelines, you’ve to find them, so are they there when you need them {I-1}*
	**Timely access (N = 1)**	*● It should be timely in terms of my time and the time it takes to read it, and to have it so I can use it {I-10}*		
**Implementation (N = 10)**				
	**-**	-	**Not enough time to use guidelines (N = 8)**	*● I don't have time to read the whole guideline {I-4}*
				*● I don't have time, I'm, I am not seeing just hypertension patients, like the cardiologist so I don't have all the time to deal with all the details and all the facts, so guidelines should be thinking about our time {FG2-P2}*
			**Too many guidelines (N = 4)**	*● There are so many of them [guidelines] and it's difficult to know which to follow because the easy ones you remember (e.g., Diabetes) because you know the ideal target;with hypertension, it tells you, this is my first choice, what should I do step by step, but I don’t think I will do that. {FG2-P2}*
				*● There are too many guidelines to keep abreast {I-2}*
			**Lack of support to implement (N = 1)**	*● We don’t have you know, enough support to help you know, explain all the things that we are doing so that the outcome is better {I-9}*
			**Resource implications of following guidelines (N = 1)**	*● One barrier is resources – I don’t think the evidence is there or the system is ready for every single male patient over the age of 50 year to get screened for prostate cancer (i.e., PSA test). I don’t think Urologists would know how much volume they would get if every single patient was screened for PSA. {I-5}*

### Objective 2: Participants’ suggestions for revising draft recommendations

Using the theory of information architecture [17,18], feedback from family physicians on the 3 draft recommendations were grouped into 3 categories: 1) Perception of the recommendation (i.e., whether physicians believed that they could or wanted to engage with the recommendation in terms of their ability to see, hear, or become aware of something through these senses). 2) Cognition of the recommendation (i.e., whether physicians understood the recommendation in terms of the mental action or process of acquiring knowledge and understanding through thoughts, experience, and the senses); and 3) Agreement on recommendations (i.e., whether participants agreed with the recommendation or the concept of guidelines in general).

Table 8 shows the feedback on the three draft recommendations provided by physicians across these 3 categories (see Additional file 2 for original recommendations). Physicians perceived the recommendations to be disorganized (N = 8), wordy (N = 6) and too long (N = 4). They suggested shortening, and visually separating or organizing the information using lists or point form in tables or a flowchart. Physicians’ understanding of the recommendations was hampered by their lack of understanding of the evidence grading system used to support the recommendations (e.g., the meaning of “Grade A, level 1”) (N = 6), its complexity (N = 4) and lack of specific information to accurately interpret statements (N = 4). To overcome these problems, physicians suggested defining vague terms, acronyms and phrases and using words familiar to physicians; using footnotes to define and specify the evidence grading system supporting recommendations; and creating organized lists, groups, tables and flowcharts to minimize complexity of the information.

**Table 8:** Family physicians’ perceptions, cognition, and agreement with 3 draft recommendations, and their suggestions for overcoming identified problems

**Category**	**Identified problem *(Number of respondents)***	**Suggestions to improve *(Number of respondents)***	**How to do it**
**Perception**	Disorganized (N = 8)	Visually separate (N = 5), organize (N = 4)	Create organized lists, groups, tables; Create flowchart
	Wordy (N = 6)	Simplify, shorten (N = 2), visually separate (N = 5)	Point form, tables
	Long (N = 4)	Simplify, shorten (N = 2); visually separate (N = 5)	Lists, tables
**Cognition**	Do not understand grading of evidence quality (N = 6)	Define grading system of evidence quality (N = 6)	Use footnotes to explain grading of evidence; Hyperlink to more information about how grading is defined
	Confusing/complex (N = 4)	Visually separate (N = 5); organize (N = 4); match the system with the real world (N = 3)	Create organized lists, groups, tables; create flowchart; use terms familiar to physicians
	Lacking information (N = 4)	Define terms and phrases (N = 4)	Define acronyms; define vague terms
**Agreement**	Not practical (lacking necessary resources, incongruent with provider and patient values) (N = 11)	Individualize (N = 4)	When formulating recommendations, consider costs, human resources, & provider & patient values
	Poor evidence (N = 6)	If the evidence is poor, simplify the recommendation (N = 4)	Do not give detailed and specific recommendations when there is weak evidence to support it
	Does not make clinical sense (no clear direction, missing information) (N = 6)	Clear and actionable language; provide more background information (N = 4)	Use active voice; include clear targets; include information about benefits and harms
	Too aggressive *(i.e., targets, intervention, monitoring)* (N = 4)	Provide background information for the recommendation (N = 8)	Acknowledge that it is a change from current practice and underscore the rationale

**Figure 3:**
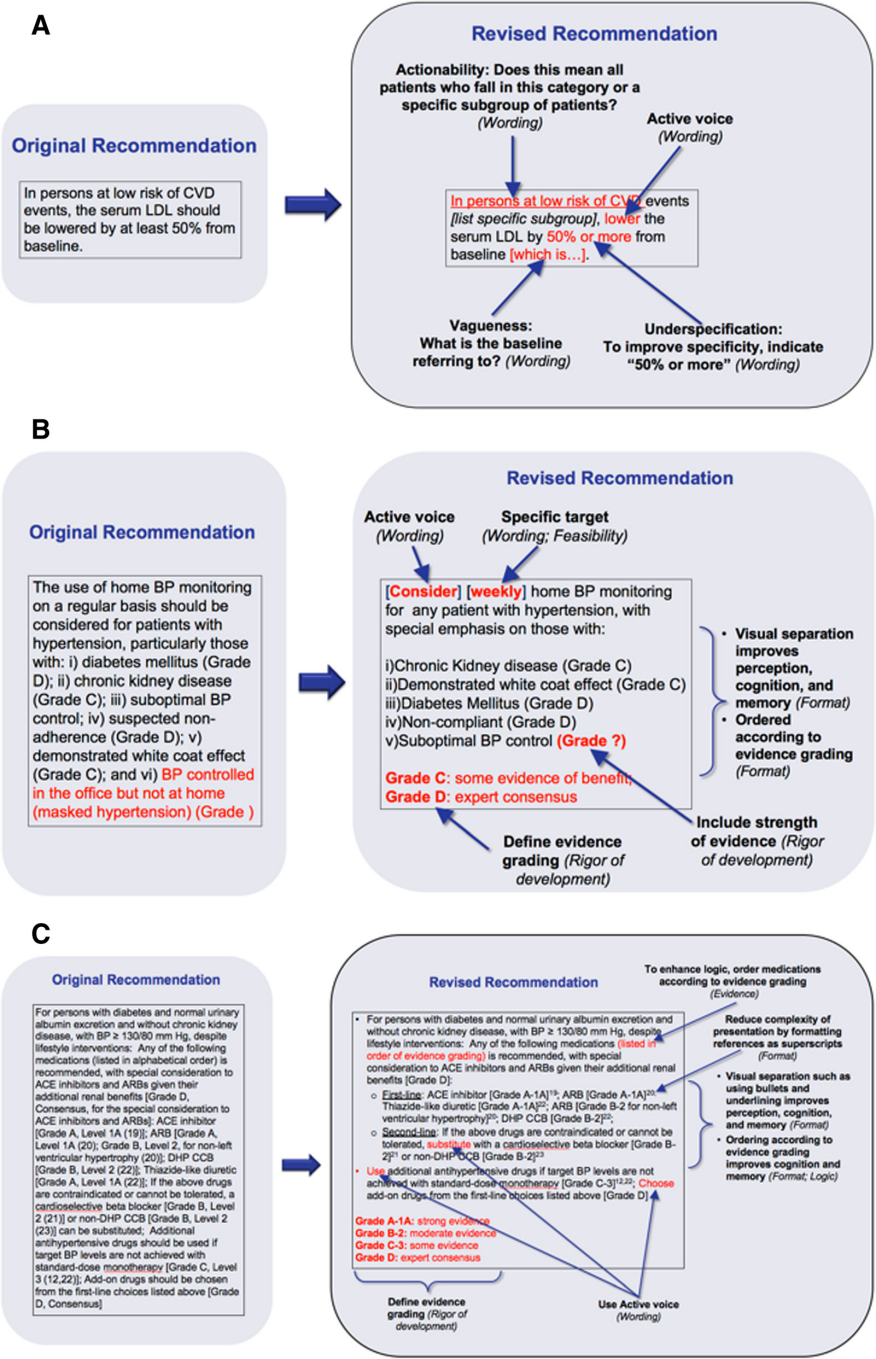
**Three draft recommendations presented to family physicians (Objective 2), depicting their perceptions of problems identified in the original recommendation and their suggestions for revising. A**. Draft recommendation 1 presented to family physicians (Objective 2), depicting their perceptions of problems identified in original recommendation and their suggestions for revising. **B**. Draft recommendation 2 presented to family physicians (Objective 2), depicting their perceptions of problems identified in original recommendation and their suggestions for revising. **C**. Draft recommendation 3 presented to family physicians (Objective 2), depicting their perceptions of problems identified in original recommendation and their suggestions for revising

There was low agreement with the 3 draft recommendation statements for several reasons. First, physicians thought that the statements were impractical (N = 11) as they did not consider the necessary resources to perform the recommendations or were incongruent with provider and patient values. To address this problem, physicians suggested individualizing recommendations by considering costs, human resources and provider and patient values in their recommendations. Second, physicians thought that the recommendations lacked clinical sense (N = 6) as there was no clear direction of action or information was missing. Suggestions were to use clear and actionable language such as active voice, the provision of more background information, to include clear targets for patient outcomes, and to include information on the benefits and harms of treatments. Another factor affecting participants’ agreement with statements was the apparent poor evidence supporting them. In such cases, physicians suggested omitting detailed and specific guidance in order to simplify recommendations. Lastly, participants perceived the recommendations to be too aggressive to apply (N = 4), that is the specified clinical targets were either not achievable or suggested interventions were more intense than what they thought their patients would prefer (e.g., being on 3 medications instead of 1 or monitoring twice a day instead of once a day). To address these issues, physicians suggested providing more background information, acknowledging that the statement represents a change from current practice, and to underscore the rationale for such changes. Figures 3A-C show each of the three original draft recommendations and the revised statements according to family physicians suggestions.

### Objective 3: Participants’ feedback on the conceptual GUIDE-IT

Three themes emerged from our discussion with participants about the conceptual guideline implememtability tool (GUIDE-IT):

Theme 1: General feedback on GUIDE-IT: Most participants believed that the tool would be useful, and that involving family physicians in the guideline development process would be important and appropriate (N = 12), but believed that these physicians should represent “regular” or community family physicians:

It should go out to family physicians, but ‘regular’ family physicians should be looking at it and you know discussing whether it is useable from their perspective {FG3-P3; FG = Focus Group; P = Participant}.

Theme 2: Strategies to involve family physicians in the guideline development process: Participants suggested several strategies to encourage the involvement of family physicians in the guideline development process. First, they suggested forming a primary care practitioner working group, which should also include patients and those involved in team-based care.

Second, participants believed that their involvement in the process would present a unique opportunity to make developers better understand what they need in the frontlines so that guidelines can become more clear, useful and less burdensome. Specifically, they suggested having a checklist to assess recommendations based on simplicity and clarity, to indicate the 5 most important points to consider, and to be able to look at how guidelines are organized:

“…for them it makes sense but for us it may not. If you were around the table, you could clarify to them what would make sense for you in your practice, and it will be faster and make it more efficient.” {FG4-P1}

However, participants also thought that there could be some downsides to family physician involvement including delays in getting guidelines out and potential barriers to sustainability given the potential costs and resources it may require to continue this process:

People get anxious about not having the latest information or how long it takes to get the guideline out (whether its up to date by the time it gets out), and if this tool will delay it significantly I think there will be resentment from the committee, even the actual guideline developers themselves and from the end users. {I-1; I = Interview}

Some participants thought that family physicians might be “drawn in” to participate purely out of interest, but most believed that some compensation would be needed for their time either in the form of a monetary incentive or through the provision of continuing medical education credits:

Another way could be is to use conferences, focus a 3-hour workshop type session where you could ask people to sign up for this and they get Mainpro-C credits. They would learn the guideline at the same time so there would be an educational component to this. {I-1}

Theme 3: Suggestions for delivering GUIDE-IT: Participants suggested that communication between guideline developers and end-users may also be facilitated through the consideration of different platforms for delivering GUIDE-IT. Some suggested a group-based live forum while most believed that an online system (or a wiki-based system) would be more efficient as this would allow family physicians to contribute to the guideline using a central location from their own computer. They also suggested that an online system would enable assessments on different platforms (e.g., smartphones and other touch screen devices), which would make assessments easier to access, and allow more focus on one idea (compared with a group situation) and thus would also *“even out the playing field”.*

## Discussion

This qualitative study revealed family physician perspectives on facilitators and barriers to guideline use across 6 categories in the context of their own practice. The most frequently described facilitators of uptake addressed guideline format, content, language and usability. In particular, family physicians expressed wanting simple, clear and uncomplicated guidelines that are easy to use or follow, relevant to primary care, and developed by a trusted and credible source. These findings are consistent with others investigating end-user perspectives of guideline implementability in the general context [19-25] and across a wide range of specific guideline topics such as cancer control [26]; urinary tract infection [27]; depression [28]; immunization [29]; infection prevention and control [30]; dementia [31]; cholesterol education [32]; and asthma [33]; and for different end users such as physiotherapists [34] and dentists [35]. Our work builds on this knowledge through the creation of a model of guideline implementability, derived exclusively from guideline end-user perspectives (Figure 2). This model represents a more in-depth understanding of the general concept of guideline implementability by showing that physicians perceive facilitators and barriers to guideline uptake almost exclusively according to intrinsic factors (i.e., those that are under the control of guideline developers). This finding strengthens the notion that optimising such factors (as perceived by their end-users) can be, and should be applied in tools addressing guideline implementability. Whether such a tool can have a positive impact, and whether it could be routinely incorporated into guideline development at minimal cost will be the focus of our future work.

Family physicians in our study also had the opportunity to respond to three draft recommendations, which highlighted problems typically encountered with guideline use (i.e., lack of understandability and applicability of recommendations). Most participants perceived draft recommendations as disorganized, wordy and long; had difficulty understanding sections they perceived as confusing or lacked information; and had a general disagreement with recommendations that were “impractical”, had poor evidence base, lacked clinical sense or if they were perceived as too aggressive (i.e., expectations for reaching proposed targets, intervention or monitoring). Family physicians were equally open to offering potential solutions to improve these recommendations, which indicates that involving end-users in their assessment during guideline development is not only feasible but shows potential for end-users to suggest more implementable recommendations. In our study, we were able to show the value of such feedback, as participants not only identified specific problems, they also provided solutions that were practical and operationalized. This level of feedback also suggests that it would be feasible to create objective criteria for assessing and revising draft guideline recommendations across various factors of guideline implementability such as those revealed in our model (Figure 2). For example, if we considered the *Wording* factor, we could apply objective rules to identify problems with the wording of draft recommendations such as its clarity (i.e., unambiguous), simplicity (minimizing multiple steps, elements or actions) and actionability (i.e., making recommendations specific and using active voice). Through identifying such factors using objective criteria across multiple intrinsic factors (i.e., wording, content, format, evidence, updating), it would be possible to generate domain scores of implementability. This would in principle allow potential problems to be identified across all domains (or in select domains). Furthermore, the same objective criteria that identified the problems in recommendations can also be used to generate solutions, and hence the potential to improve the overall implementibility of the guideline.

Family physicians iterated the importance of their involvement in guideline development to ensure that developers better understand their needs, particularly for frontline practice. However, they suggested that those tasked with assessing the implementability of draft recommendations should comprise mostly of non-academic or “regular” (i.e., community) family physicians with no particular expertise or interest in a specific disease area. Since “regular” physicians are rarely engaged in guideline development, participants thought that their involvement would maximize the objectivity of GUIDE-IT assessments and the potential that recommendations vetted through GUIDE-IT will be more applicable. Such engagement can also identify problems with the applicability and flexibility of guidelines [36,37], which in turn will foster greater adherence to guidelines and a sense of ownership [27]. Our study participants also thought it was important to balance the potential benefits with barriers such as the potential for delays in completing guidelines resulting from this involvement (i.e., completing assessments), and the increased costs and resources that would be needed to recruit physicians and sustain the process. Furthermore, even though most national guideline development groups and organizations advocate for the involvement of physicians and patients during the development process (e.g., Guideline International Network [GIN], Canadian Task Force on Preventive Health Care [CTFPHC]; Institute of Medicine [IOM]; and the National Institute for Health and Care Excellence [NICE]), most do not provide information about which specific end-users should be involved, how they should be selected or recruited, when they should be involved and how [38-41].

Lastly, participants had the opportunity to provide feedback on the conceptual GUIDE-IT tool. They suggested that an individualized online platform would be the most practical forum to deliver GUIDE-IT assessments. They believed that a computerized system would provide more flexible participation and easier access to completing recommendation assessments, including the delivery of GUIDE-IT using diverse platforms such as applications for smartphones and other touch screen devices.

One of the strengths of our study was that we addressed the potential limitations inherent to qualitative studies such as threats to internal validity (i.e., credibility), external validity (i.e., transferability) and reliability (dependability) [42]. We addressed potential threats to credibility by pilot testing the interview questions to ensure that they were well understood, and to use experienced moderators to lead discussions. As with other qualitative studies, our findings are not generalizable beyond the sample of family physicians that participated in our study. However, we used a mixed sampling strategy (random and purposive) in an attempt to identify a wide scope of family practice. Furthermore, we provided detailed information about the family practices and physicians to enhance the transferability of our findings. To limit the potential of biases that may be introduced by investigators with respect to the dependability and confirmability of our work, we standardized procedures, methods, and analysis strategies across all interviews. Furthermore, sessions were planned so that physicians were prompted about their perceptions of the facilitators and barriers to guideline implementability in the context of their own practice before introducing the conceptual design of our GUIDE-IT tool, thereby avoiding any opportunity for contamination. Finally, we believe that our findings are transferable to community and academic family practices in the Toronto general area.

Next steps include applying the findings of this study (in consultation with information technologists and human factors engineers) to transform the conceptual GUIDE-IT design into a functioning prototype. This will involve creating GUIDE-IT as an online system that will be accessible through multiple applications and devices, and the development of a systematic, operationalized, and sustainable approach for selecting and recruiting family physicians for completing GUIDE-IT assessments. For example, we will attempt to optimise strategies for family physician involvement in the use of GUIDE-IT by balancing identified resource and time burdens that may delay guideline production. Once the prototype is finalized, we will test it with guideline developers and all relevant end-users (all physicians who use guidelines) to determine its usability for assessing the implementability of guidelines. Next, we will conduct a randomized trial to determine the impact of GUIDE-IT on improving guideline recommendations and its impact on subsequent guideline use by family physicians.

## Conclusions

Our study identified facilitators and barriers from the perspective of guideline end users that will be used to transform our GUIDE-IT tool into a functional prototype. Our findings build on current knowledge of guideline implementability by showing that family physicians perceive guideline uptake mostly according to factors that are in the control of guideline developers. The study also identified numerous intrinsic barriers that could be minimized during guideline development if developers elicit them from potential end users. Finally, it highlights the importance of end user feedback and the willingness of end users to provide feedback in order for developers to produce more implementable products. Characterization of facilitators and barriers can be used to optimize tools and interventions such as GUIDE-IT to overcome barriers and improve guideline uptake.

## Competing interests

The authors have no competing interests to declare.

## Authors’ contributions

All authors participated in the design and development of the protocol. MK and EE conducted the interviews, and MK, LH, AG, and AC analysed the data. MK drafted the manuscript, and all authors read and approved the final manuscript.

## Pre-publication history

The pre-publication history for this paper can be accessed here:

http://www.biomedcentral.com/1471-2296/15/19/prepub

## Supplementary Material

Additional file 1 Semi-structured Interview Guide. Click here for file

Additional file 2 Draft guideline recommendations used to elicit feedback from family physician participants during interview sessions*. Click here for file
