# Supplementary material for: The development of a guideline implementability tool (GUIDE-IT): a qualitative study of family physician perspectives
Source: BMC Fam Pract. 2014 Jan 29;15:19. doi: 10.1186/1471-2296-15-19 (PMC4016596; doi:10.1186/1471-2296-15-19)
Supplement: Additional file 1 — Semi-structured Interview Guide. [file 1471-2296-15-19-S1.doc]

**Appendix A**

Semi-structured Interview Guide

**PART I: Perceptions of guidelines (20-30 minutes)**

1. Do you use guidelines in your practice? Why/why not?

- *If no, what other resources do you use in practice?*

1. When and how do you use guidelines in practice?

- *At the point of care? Other times?*
- *What is the situation? Can you give an example?*
- *How often do you use it per week and for what situations?*

1. What do you find useful about guidelines? *(Facilitators)*
2. What do you find difficult about using guidelines? *(Barriers)*
3. In general, what information do you want to see in guidelines?

- *How should this information be represented?*

1. What do you think are the qualities of guidelines that make it easier for you to decide how to manage a patient? What qualities of guidelines would make it more difficult? *(Attributes)*
2. What do you think guideline developers should take into consideration when they write and develop guidelines for family physicians?
3. Do you think family physicians should be part of the guideline development process? How would this work?

**PART II: Perceptions of guideline recommendations (20-30 minutes)**

*Present PPT slides of draft recommendations (as many as possible in time available) from the C-CHANGE draft recommendations and ask the following questions:*

1. What do you think the recommendation is saying? Do you understand it? If no, what aspects do you find difficult?
2. What would you do with this recommendation in practice?
3. Would you change this recommendation? Why/why not? How would you change it?

**PART III: Perceptions of a guideline implementability tool (20-30 minutes)**

1. Evidence indicates that physicians are not using guidelines. If you had to develop a tool that would improve this situation (to help physicians use guidelines more), what would this tool look like?

*Present our framework/tool and ask the following questions:*

1. What do you think of this tool? *(GUIDE-IT)*
2. What barriers do you foresee to applying this tool? *(Barriers to the process)*
3. We believe that it is important to establish a relationship between guideline developers and end-users during the guideline development process, and we would like this to be one of the components of our tool. Do you have any suggestions how we could get family physicians to be involved in the development of guidelines?
4. Do you have any other suggestions on how we could improve recommendations before guidelines are finalized?
5. Do you have any other questions?
