# Supplementary material for: The development of a guideline implementability tool (GUIDE-IT): a qualitative study of family physician perspectives
Source: BMC Fam Pract. 2014 Jan 29;15:19. doi: 10.1186/1471-2296-15-19 (PMC4016596; doi:10.1186/1471-2296-15-19)
Supplement: Additional file 2 — Draft guideline recommendations used to elicit feedback from family physician participants during interview sessions*. [file 1471-2296-15-19-S2.doc]

**Appendix B**

Draft guideline recommendations used to elicit feedback from family physician participants during interview sessions*

| **Draft recommendation 1** | In persons at low risk of CVD events, the serum LDL should be lowered by at least 50% from baseline. |
| --- | --- |
| **Draft recommendation 2** | The use of home BP monitoring on a regular basis should be considered for patients with hypertension, particularly those with: i) diabetes mellitus (Grade D); ii) chronic kidney disease (Grade C); iii) suboptimal BP control; iv) suspected non-adherence (Grade D); v) demonstrated white coat effect (Grade C); and vi) BP controlled in the office but not at home (masked hypertension) (Grade ?). |
| **Draft recommendation 3** | For persons with diabetes and normal urinary albumin excretion and without chronic kidney disease, with BP ≥ 130/80 mm Hg, despite lifestyle interventions: Any of the following medications (listed in alphabetical order) is recommended, with special consideration to ACE inhibitors and ARBs given their additional renal benefits [Grade D, Consensus, for the special consideration to ACE inhibitors and ARBs]: ACE inhibitor [Grade A, Level 1A (19)]; ARB [Grade A, Level 1A (20); Grade B, Level 2, for non-left ventricular hypertrophy (20)]; DHP CCB [Grade B, Level 2 (22)]; Thiazide-like diuretic [Grade A, Level 1A (22)]; If the above drugs are contraindicated or cannot be tolerated, a cardioselective beta blocker [Grade B, Level 2 (21)] or non-DHP CCB [Grade B, Level 2 (23)] can be substituted; Additional antihypertensive drugs should be used if target BP levels are not achieved with standard-dose monotherapy [Grade C, Level 3 (12,22)]; Add-on drugs should be chosen from the first-line choices listed above [Grade D, Consensus]. |

*Draft guideline recommendations were provided by the Canadian Cardiovascular Harmonization of National Guidelines Endeavour (C-CHANGE) group.
